# Dust Exposure Associations with Lung Function among Ethiopian Steel Workers

**DOI:** 10.5334/aogh.2422

**Published:** 2019-01-22

**Authors:** Feyisa Girma, Zeyede Kebede

**Affiliations:** 1 Center for Environmental Science, Addis Ababa University, ET; 2 World Health Organization Country Office, Addis Ababa, ET

## Abstract

**Background:**

Booming industrial development in Ethiopia, including a growing steel industry, may result in increased prevalence of pulmonary conditions. In this study, we evaluated steel workers’ exposure to dust as well as its potential impact on lung function.

**Methods:**

Cross-sectional study of 75 steel workers in Ethiopia, interviewed from April to June 2015. We obtained information on respiratory symptoms and personal protective equipment use via interview and conducted spirometry testing to assess lung function. Dust samples were collected from different factory sections. Correlation analyses were used to assess associations between variables.

**Results:**

Maximum dust levels were recorded in the induction furnace, where both galvanized and non-galvanized metals are melted. Steel factory workers with higher levels of particulate matter exposure had increased rate of respiratory symptoms (r = 0.96). Forced vital capacity values showed a strong negative correlation with numbers of years at work (r = –0.86, p = 0.03) and responders age (–0.85, p = 0.49) and weak negative correlation with level of particular matter (PM) (r = –0.02, p = 0.07). Similarly, forced expiratory volume in 1 second was strongly negatively correlated with the number of years of exposure (r = –0.82, p = 0.05) and workers age (r = –0.85, p = 0.08) and weakly negatively correlated with PM level (r = 0.25, p = 0.67).

**Conclusions:**

Occupational exposure continues to be a major problem among steel factory workers in Ethiopia and is associated with lung function abnormalities. Ensuring the availability of proper personal protective equipment, regular factory inspections, and training may help mitigate the impact of occupational exposures among these workers.

## Introduction

Most steel manufacturing processes in Ethiopia are not automated and therefore workers are directly involved in many process and tasks. For example, scrap metals segregation, collection and addition to furnaces are manually performed. These activities lead to high levels of exposure to dust, fumes, heavy metals, smoke, hot materials and other toxins [1,2]. Consequently, work-related environmental factors may have a major impact on the health and well-being of steel workers.

High prevalence of occupational respiratory problems among metal workers are well documented [3,4]. Additionally, prior studies have demonstrated reduction in lung function due to exposure to high respirable dust levels in steel factory workers [1,5,6]. While data from other countries suggest a strong exposure-disease relationship, the negative health impacts of dust exposures among Ethiopian steel factory workers is unknown. Despite the high levels of exposure to metal dust without appropriate availability of personal protective equipment and limited safety training. In this study, we assessed whether exposure to dust is associated with lung function decrease among workers in steel factories in Ethiopia.

## Methods

The study was conducted in a steel factory located in Addis Ababa, Ethiopia. The factory, opened in 1960, manufactures reinforcement bars, fencing nets, bed spring nets, nails, black tie wire and bartended wire.

We conducted a cross sectional study from April to June, 2015. A steel factory in Addis Ababa was selected for the site of the study based on accessibility and representativeness criteria. Simple random-sampling was then used to select study participants among workers in different sections of this steel factory. The study was limited to workers in the production and work shop section of the factory. Workers with known health problems or prior injuries were excluded from the study.

## Data collection

Suspended particulate matter (PM) at various sections of the steel factory were measured using a dust sampler RAM4™, model DR-4000 (Thermo Anderson, US). The PM sampling equipment was placed one meter above ground in factory areas where the workers reported spending most of their time. Sapling was conducted during active production periods. The sampler device was calibrated at the start of each collection period following the manufacturer’s instructions. PM was classified in to course (PM_10_) and fine (PM_2.5_) based on aerodynamic particle size.

Lung function testing was carried out by using Spiro Lab III (Medical International Research, Italy). Measurements of forced vital capacity (FVC), forced expiratory volume in one second (FEV_1_), FEV_1_/FVC ratio, were performed while participants were sitting in the upright position, half an hour after they started their job. Measurements were repeated three times by a trained technician following standardized procedures; the best results were used for analyses [7,8]. The standing height and weight of each subject was measured before lung function testing and used to calculate percent predicted values. We collected data about participants’ working area, respiratory health symptoms and use of personal protective equipment using standardized questionnaire.

## Statistical Analysis

Descriptive statistics are presented using means and standard deviations or proportions, as appropriate. The correlation between workers’ occupational exposures and lung function measures were assessed using the Spearman correlation coefficient. Analyses were conducted with SPSS version 20 (IBM Corp, Armonk, New York) using two-sided p-values. The study was approved by the Center for Environmental Science’s ethical committee of Addis Ababa University; verbal consent was obtained from all participants.

## Results

### Occupational Exposure to PM

The maximum dust levels were recorded in the induction furnace, where both galvanized and non-galvanized metals are melted. In the induction furnace when non-galvanized iron was melted, mean PM level was 1,025.0 ± 0.4 μg/m^3^ but increased to 2,061.1 ± 306.7 μg/m^3^ during active periods. In the second stage dice area mean PM level was 308.5 ± 24.4 μg/m^3^; in the nail production area, the mean PM level was 236.3 ± 19.3 μg/m^3^ and in the nail cleaning area the mean PM level was 153.7 ± 67.1 μg/m^3^ (Table 1). The highest mean course PM_10_ was found in the first stage dice area (4,311.0 ± 1, 80.5 μg/m^3^). As with course PM, the highest mean fine PM_2.5_ was also recorded in first stage dice 2,629.3 ± 183.5 μg/m^3^ followed by the induction furnace when galvanized metals were melted 2,159.3 ± 3, 22.7 μg/m^3^ (Table 2).

**Table 1:** Particular matter levels in different sections of the steel factory.

Factory Section	Minimum level in μg/m^3^	Maximum level in μg/m^3^	Mean level in μg/m^3^

Induction furnace area when Non-galvanized iron melted	115.2	8,754.6	1,025.0 ± 0.4
Induction furnace when galvanized iron melted	152.0	17,821.2	2061.1 ± 306.7
First stage dice area	862.6	6,523.7	2,927.0 ± 1,782.4
Second stage dice area	109.0	1,088.0	308.5 ± 24.4
Nail production	110.1	2,033.5	236.3 ± 19.3
Nail cleaning	597	84.6	153.7 ± 67.1

**Table 2:** PM_2.5_ and PM_10_ mean levels and diameter in different section of the steel factory.

Factory section	Mean PM_2.5_ level in μg/m^3^	Mean PM_2.5_ diameter in μm	Mean PM_10_ level in μg/m^3^	Mean PM_10_ diameter in μm

Induction furnace area when non-galvanized iron melted	853.4 ± 1,537.9	1.4 ± 0.5	156.0 ± 21.7	2.7 ± 0.6
Induction furnace when galvanized iron melted	2,159.3 ± 322.7	1.2 ± 0.5	1,393.2 ± 1,541.6	3.3 ± 0.7
First stage dice area	2,629.3 ± 183.5	1.9 ± 1.4	4,311.0 ± 180.5	3.4 ± 0.7
Second stage dice area	341.3 ± 239.9	1.2 ± 0.5	494.5 ± 210.6	3.3 ± 0.6
Nail production	220.1 ± 224.8	1.8 ± 0.4	252.5 ± 158.1	3.1 ± 0.5
Nail cleaning	151.1 ± 64.0	1.2 ± 0.4	203.8 ± 120.2	2.7 ± 0.2

PM_2.5_ denotes: Fine particulate matte. PM_10_ denotes: Course particulate matter.

### Workers Characteristics and Self-reported Respiratory Symptoms

A total of 161workers were enrolled in the study. Of these, 76 workers were involved in nail production, 27 worked in both induction and arc furnace, 48 in the rolling mill and 10 in the die room. On average, these workers were exposed to different levels of PM for 8 hours per day. Only one participant (1%) reported being an active smoker. Twenty-one (28%) of respondents did not regularly use personal protective equipment. Pulmonary symptoms reported by workers included breathing difficulties, (n = 19, 25%), frequent wheezing (n = 24, 32%) and sneezing (n = 29, 38%; Table 3). Most workers (n = 65, 86%) developed respiratory symptoms after they started working in the steel factory.

**Table 3:** Self-reported behaviors, environmental exposures and respiratory symptoms among participating workers.

Characteristic	Yes		No	

	Number	Percentage	Number	Percentage

Alcohol use	45	60	30	40
Tobacco smoking	1	1	74	99
Personal protective equipment use	54	72	21	28
Breathing difficult	19	25	56	75
Wheezing	24	32	51	68
Sneezing	29	39	46	61

### Lung Function according to Work Location

There was substantial variability in the number of years of exposure among workers in different factory areas. Nail production workers had the longest work history (mean 22.9 ± 12.2 years, range 8 to 23 years). Mean working history for other groups was: 17.7 ± 14.2 years (range 2 to 38 years) for rolling mill workers, 13.9 ± 12.0 years (range 1 to 35 years) for dice machine workers, and 3.6 ± 2.7 years (range 1 to 38 years) for induction furnace workers.

Mean FVC in induction furnace workers was 3.31 ± 0.55 liters, mean FEV_1_ was 2.79 ± 0.55 liters and mean FEV_1_/FVC ratio was 81.5 ± 14.9%. In the rolling mill mean areas workers’ FVC was 2.48 ± 0.59 liters, FEV_1_was 2.38 ± 0.06 liters and FEV_1_/ FVC was 82.39 ± 30.47%, in nail production areas workers’ FVC was 2.45 ± 0.03 liters, FEV_1_ was 2.10 ± 0.90 liters and FEV_1_/ FVC was 80.53 ± 22.04% and in die machines area mean workers’ FVC was 2.37 ± 0.55, FEV_1_ was 2.11 ± 0.55 liters and FEV_1_/ FVC was 88.40 ± 10.4%.

FVC values showed a strong negative correlation with numbers of years at work (r = –0.86, p = 0.03) and responders age (–0.85, p = 0.49) and weak negative correlation with level of PM (r = –0.02, p = 0.07). Similarly, FEV_1_was strongly negatively correlated with the number of years of exposure (r = –0.82, p = 0.05) and workers age (r = –0.85, p = 0.08) and weakly negatively correlated with PM level (r = 0.25, p = 0.67) while FEV_1_/ FVC strongly negatively correlated with PM level (r = 0.74, p = 0.43).

## Discussion

In this study, we evaluated levels of PM exposure and its relationship with lung function among a cohort of steel workers in an Ethiopian steel factory. We found high levels of exposure to PM among these workers and a strong correlation of lung function abnormalities with the extent of metal dust exposure. These findings suggest there is the need for public health work to prevent toxic exposures among these workers and subsequent impairment in lung function.

World Health Organization guidelines provide strict guidelines about maximal levels of back ground concentration of toxin in the workplace. Proposed value for PM_2.5_ mean annual exposure is 10μg/m^3^ and 25 μg/m^3^ for 24-hour mean; suggested threshold for PM_10_ values are 20 μg/m^3^ for annual mean and 50 μg/m^3^ for 24-hour mean [9]. Similarly, the American Conference of Governmental Industrial Hygienists guidelines released in 2009 recommend a threshold limit value of 3,000μg/m^3^ for PM_2.5_ and the United States Occupational Safety and Health Administration has established an 8-hour time weighted average limit of 15 mg/m^3^, measured as total particulate and the 5 mg/m^3^ limit for respirable particle [10]. Except for the nail production and nail cleaning areas, the 8-hour time weighted average PM_2.5_ was well above these threshold values, suggesting potential risks for the health of these workers. Thus, public health measures are needed to curtail potential toxic exposures among steel workers in Ethiopia. Consistent with prior studies our results also showed that many steel factory workers experience respiratory symptoms [3,4,11,12,13]. However, we found larger defects in lung function compared to those reported by Singh et al. among similar workers [5]. Many workers on our study showed signs of reduced FEV_1_ which may indicate obstructive lung disease. In addition, other workers had reduced FVC values suggesting restrictive lung problems which may be related to the long-term exposure to PM documented in our study.

Consistent with the literature, the number of years of exposure was associated with reduced FEV_1_, FVC and the FEV_1_/FVC measurements [3,14]. Combination of obstructive and restrictive patterns are frequently observed in lungs of steel workers [5,15,16,17,18] and equally worrisome is the fact that long-term exposure to high levels of PM, particularly PM_2.5_, is an independent risk factor for lung cancer [9,16,18,19,20,21,22].

## Conclusions

We found that many steel workers in an Ethiopian steel factory were exposed to high levels of PM without using adequate personal protective equipment. Many of these workers also reported high prevalence of respiratory symptoms and/or developed lung function abnormalities after they were employed in the factory. Given that fume and dust spreads from the induction furnace to the rest of the steel plant, using barriers such as wood, metals, or chimney on the furnace to enclose certain areas is highly recommended. Furthermore, there should be occupational health and safety training and regular inspection in place.
